# Crystal structure and Hirshfeld surface analysis of tetra­aqua­bis­(isonicotinamide-κ*N* ^1^)nickel(II) fumarate

**DOI:** 10.1107/S2056989018013580

**Published:** 2018-10-02

**Authors:** Sevgi Kansiz, Irina A. Golenya, Necmi Dege

**Affiliations:** a Ondokuz Mayıs University, Faculty of Arts and Sciences, Department of Physics, 55139, Kurupelit, Samsun, Turkey; b Taras Shevchenko National University of Kyiv, Department of Chemistry, 64, Vladimirska Str., Kiev 01601, Ukraine

**Keywords:** crystal structure, fumaric acid, isonicotinamide, nickel(II), Hirshfeld surface

## Abstract

The reaction of NiCl_2_ with fumaric acid and isonicotinamide in a basic solution produces the title complex, [Ni(C_6_H_6_N_2_O)_2_(H_2_O)_4_](C_4_H_2_O_4_). The nickel(II) ion of the complex cation and the fumarate anion are each located on an inversion centre. The Ni^II^ ion is coordinated octa­hedrally by four water O atoms and two N atoms of isonicotinamide mol­ecules. The fumarate anion is linked to neighbouring complex cations *via* O_water_—H⋯O_fumarate_ hydrogen bonds. In the crystal, the complex cations are further linked by O—H⋯O, N—H⋯O and C—H⋯O hydrogen bonds, forming a three-dimensional supra­molecular architecture. Hirshfeld surface analysis and two-dimensional fingerprint plots were used to analyse the inter­molecular inter­actions present in the crystal and indicate that the most important contributions for the crystal packing are from H⋯O/O⋯H (41.8%), H⋯H (35.3%) and H⋯C/C⋯H (10.2%) inter­actions.

## Abstract 2

In the crystal, the Ni^II^ complex cation and fumarate anion are located on individual inverse centers and linked *via* O—H⋯O, N—H⋯O and C—H⋯O hydrogen bonds.

## Chemical context   

Metal complexes of biologically important ligands are sometimes more effective than the free ligands. Many transition and heavy metal cations play an important role in the biological processes involved in the formation of vitamins and drug components. An important element for biological systems is nickel and nickel complexes have biological activities including anti­epileptic, anti­microbial, anti­bacterial and anti­cancer activities (Bombicz *et al.*, 2001 ▸). Di­carb­oxy­lic acid ligands have been utilized primarily in the synthesis of a range of metal complexes. Di­carb­oxy­lic acids such as fumaric acid and amides have been particularly useful in creating many supra­molecular structures (Pavlishchuk *et al.*, 2011 ▸; Ostrowska *et al.*, 2016 ▸), in particular isonicotinamide with a variety of carb­oxy­lic acids (Vishweshwar *et al.*, 2003 ▸; Aakeröy *et al.*, 2002 ▸).
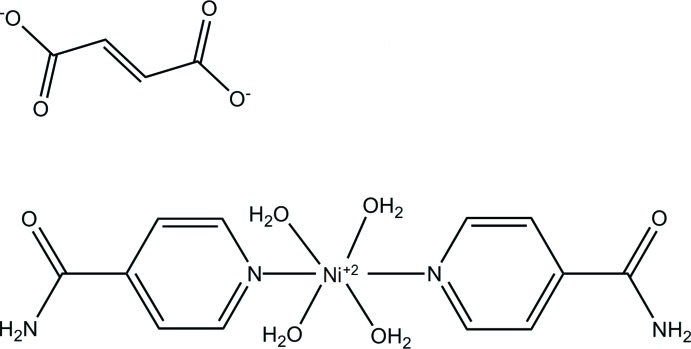


We have prepared a new Ni^II^ complex, tetra­aqua­bis(isonicotinamide-κ*N* ^1^)nickel(II) fumarate, and determined its structure by single crystal X-ray diffraction. In addition, Hirshfeld surface analysis and fingerprint plots were used to understand the inter­molecular inter­actions in the crystal structure.

## Structural commentary   

**Figure 1:**
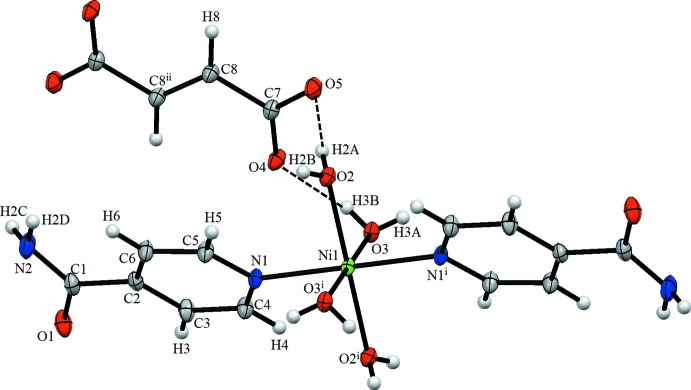
The mol­ecular structure of the title compound, showing the atom labelling. Displacement ellipsoids are drawn at the 20% probability level. [Symmetry codes: (i) −*x* + 1, −*y* + 1, −*z* + 1; (ii) −*x* + 1, −*y* + 1, −*z*.]

The mol­ecular structure of the title complex is illustrated in Fig. 1 ▸. The nickel(II) ion is octa­hedrally coordinated to four water O atoms and two N_pyridine_ atoms of isonicotinamide mol­ecules. The values of the Ni—O_water_ and Ni—N_pyridine_ bond lengths and the bond angles involving atom Ni1 (Table 1 ▸) are close to those reported for similar nickel(II) complexes (Krämer *et al.*, 2002 ▸; Bora & Das, 2011 ▸; Moroz *et al.*, 2012 ▸).

## Supra­molecular features   

**Figure 2:**
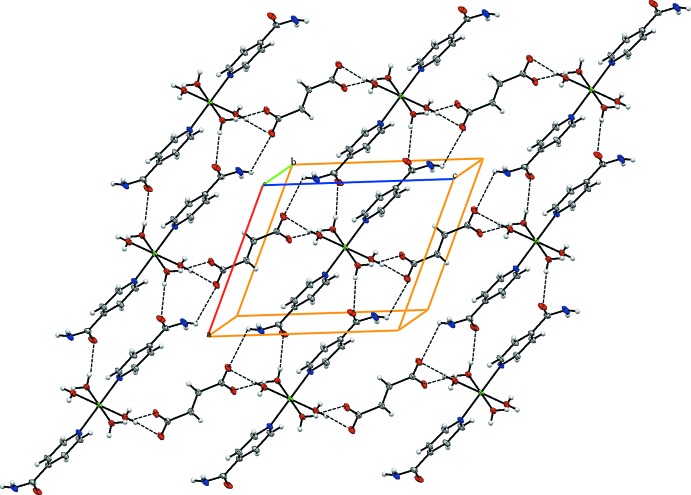
A view of the crystal packing of the title compound. Dashed lines indicate hydrogen bonds.

In the crystal, each O atom of the fumarate dianion is linked to a water H atom *via* O—H⋯O hydrogen bonds, forming chains along the *c*-axis direction (Table 2 ▸, Fig. 2 ▸).

The fumarate anions and complex cations are linked by O—H⋯O hydrogen bonds; the complex cations also inter­act with each other through O—H⋯O, N—H⋯O and C—H⋯O hydrogen bonds, forming a three-dimensional supra­molecular architecture.

## Database survey   

A search of the Cambridge Structural Database (CSD, version 5.39, update of May 2018; Groom *et al.*, 2016 ▸) revealed the structures of four similar tetra­aqua­bis­(isonicotinamide-κ*N* ^1^)nickel(II) complexes with different counter-anions *viz.* bis­(4-formyl­benzoate) dihydrate (HUCLAT; Hökelek *et al.*, 2009 ▸), bis­(3-hy­droxy­benzoate) tetra­hydrate (GANZAY; Zaman *et al.*, 2012 ▸), bis­(thio­phene-2,5-di­carboxyl­ate) dihydrate (NETQIO; Liu *et al.*, 2012 ▸) and naphthalene-1,5-di­sulfonate tetra­hydrate (TESDEC; Lian, 2012 ▸). In all four complexes, the cation possesses inversion symmetry with the nickel ion being located on a centre of symmetry. The Ni—O_water_ bond lengths vary from 2.044 to 2.086 Å, while the Ni—N_pyridine_ bond lengths vary from 2.075 to 2.098 Å. In the title complex, the cation also possesses inversion symmetry and the Ni—O_water_ bond lengths [2.0812 (15) and 2.0537 (16) Å] and the Ni—N_pyridine_ bond length [2.1075 (18) Å] fall within these limits.

## Hirshfeld surface analysis   

**Figure 3:**
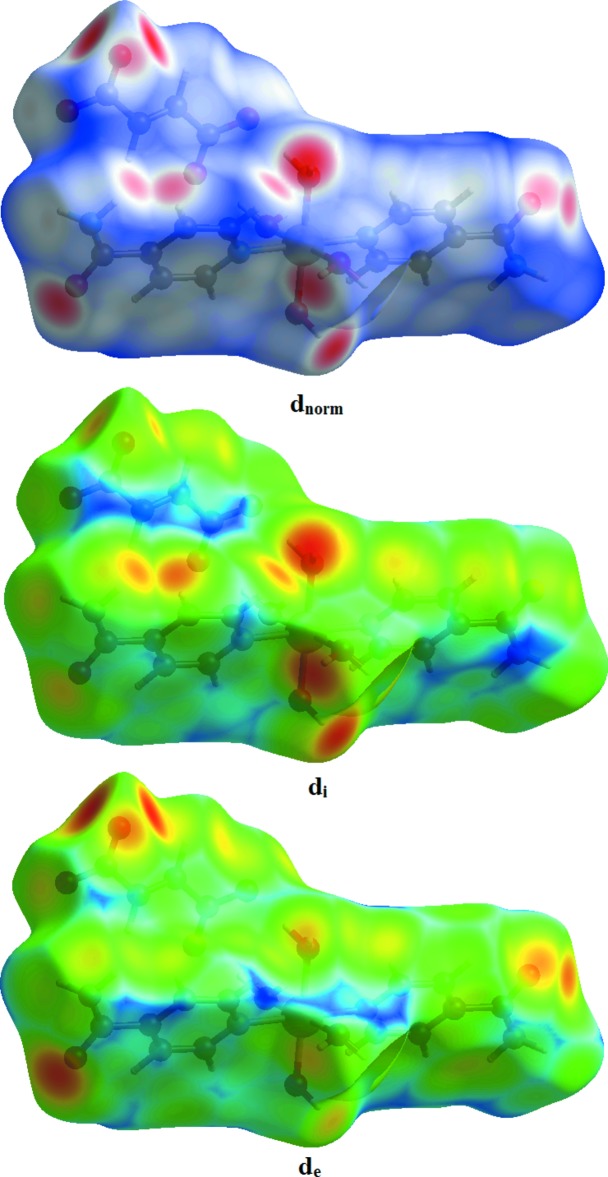
The Hirshfeld surface of the title compound mapped over *d*_norm_, *d*_i_ and *d*_e_.

**Figure 4:**
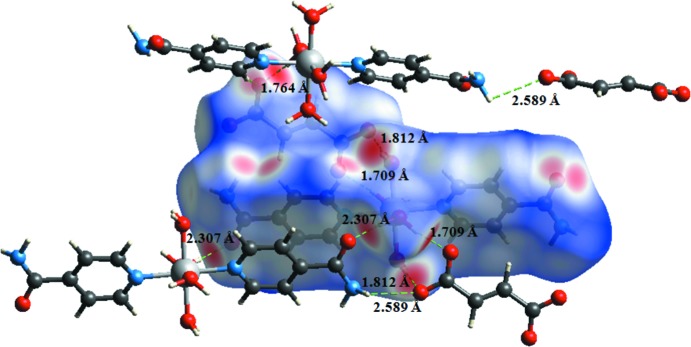
The Hirshfeld surface mapped over *d_norm_* to visualize the intra­molecular and inter­molecular inter­actions in the title compound.

**Figure 5:**
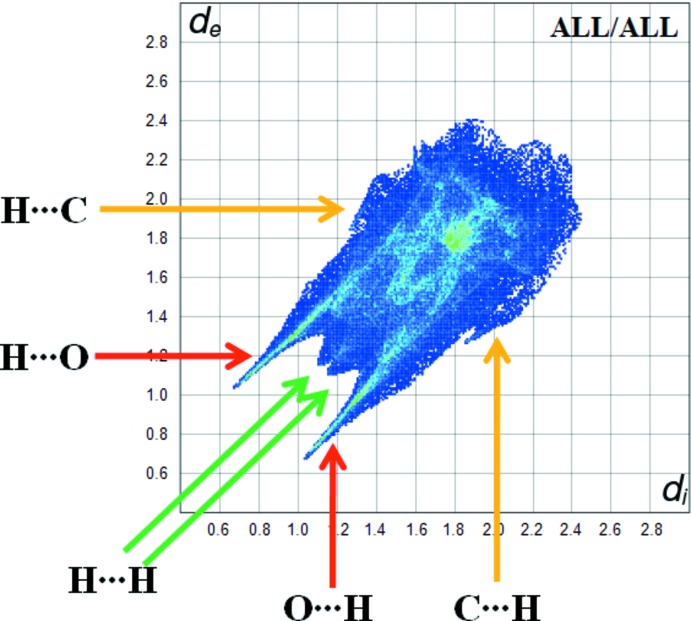
A fingerprint plot of the title compound.

**Figure 6:**
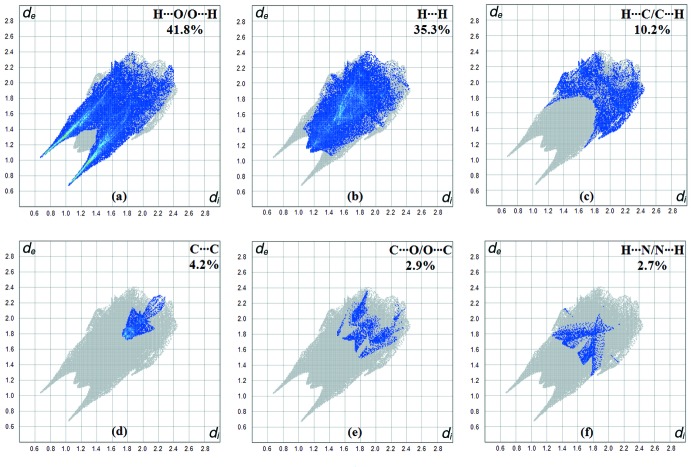
(*a*) H⋯O/O⋯H, (*b*) H⋯H, (*c*) H⋯C/C⋯H, (*d*) C⋯C, (*e*) C⋯O/O⋯C and *(f*) H⋯N/N⋯H contacts in the title complex, showing their percentage contributions to the Hirshfeld surface.

*Crystal Explorer17.5* (Turner *et al.*, 2017 ▸) was used to investigate the Hirshfeld surfaces and to analyse the inter­actions in the crystal. The Hirshfeld surfaces mapped over *d*_norm_, *d*_i_ and *d*_e_ are shown in Fig. 3 ▸. Red spots indicate the contacts involved in strong hydrogen bonds and inter­atomic contacts (Gümüş *et al.*, 2018 ▸; Sen *et al.*, 2018 ▸; Kansız & Dege, 2018 ▸); those in Fig. 3 ▸ correspond to the near-type H⋯O contacts resulting from C—H⋯O, O—H⋯O and N—H⋯O hydrogen bonds. The Hirshfeld surfaces were obtained using a standard surface (high) resolution with the three-dimensional *d_norm_* surfaces mapped over a fixed colour scale of −0.701 (red) to 1.286 (blue) a.u. The red spots in Fig. 4 ▸ correspond to the near-type H⋯O contacts resulting from O—H⋯O and N—H⋯O hydrogen bonds. Fig. 5 ▸ shows the two-dimensional fingerprint plot of the sum of the contacts contributing to the Hirshfeld surface represented in normal mode. In Fig. 6 ▸ *a*. the two symmetrical points at the top, bottom left and right with *d*_e_ + *d*_i_ = 1.7 Å indicate the presence of H⋯O/O⋯H (41.8%) contacts. Fig. 6 ▸ *b* shows the two-dimensional fingerprint plot of the (*d*_i_, *d*_e_) points associated with hydrogen atoms and is characterized by an end point that points to the origin and corresponds to *d*_i_ = *d*_e_ = 1.08 Å, which indicates the presence of the H⋯H contacts (35.3%). Fig. 6 ▸ *c* shows the contacts between the carbon atoms inside the surface and the hydrogen atoms outside the surface of Hirshfeld and *vice versa* (H⋯C/C⋯H) and has two symmetrical wings on the left and right sides (10.2%). C⋯C (4.2%), C⋯O/O⋯C (2.9%) and H⋯N/N⋯H (2.7%) contacts also contribute to the Hirshfeld surface.

## Synthesis and crystallization   

A solution of NaOH (52 mmol, 2.07 g) was added to an aqueous solution of fumaric acid (26 mmol, 3 g) under stirring. A solution of NiCl_2_·6H_2_O (25 mmol, 6.14 g) in methanol was then added. The mixture was heated at 353 K for 30 min. and then the blue mixture was filtered and left to dry at room temperature. The reaction mixture (0.88 mmol, 0.20 g) was dissolved in methanol and added to a ethanol solution of isonicotinamide (1.76 mmol, 0.21 g). The mixture was heated at 353 K for 60 min. under stirring and the resulting suspension was filtered and left to crystallize for three weeks at room temperature. The title compound was obtained as a blue solid and contained crystals suitable for X-ray diffraction analysis.

## Refinement   

Crystal data, data collection and structure refinement details are summarized in Table 3 ▸. The water and NH_2_ hydrogen atoms were located from difference-Fourier maps and freely refined. The C-bound H atoms were positioned geometrically and refined using a riding model: C—H = 0.93–0.97 Å with *U*_iso_(H) = 1.2*U*_eq_(C).

## Supplementary Material

Crystal structure: contains datablock(s) I. DOI: 10.1107/S2056989018013580/qm2126sup1.cif

Structure factors: contains datablock(s) I. DOI: 10.1107/S2056989018013580/qm2126Isup2.hkl

CCDC reference: 1579677

Additional supporting information:  crystallographic information; 3D view; checkCIF report

## Figures and Tables

**Table 1:** Selected geometric parameters (Å, °)

Ni1—O3	2.0537 (16)	Ni1—N1	2.1075 (18)
Ni1—O2	2.0812 (15)		
			
O3—Ni1—O2	92.00 (7)	O3—Ni1—N1	86.97 (7)
O3—Ni1—O2^i^	88.00 (7)	O2—Ni1—N1	92.05 (6)
O3^i^—Ni1—N1	93.03 (7)	O2^i^—Ni1—N1	87.95 (7)

Symmetry code: (i) 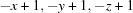.

**Table 2:** Hydrogen-bond geometry (Å, °)

*D*—H⋯*A*	*D*—H	H⋯*A*	*D*⋯*A*	*D*—H⋯*A*
O3—H3*B*⋯O4	0.80 (3)	1.89 (3)	2.678 (2)	169 (3)
O2—H2*A*⋯O5	0.78 (3)	2.01 (3)	2.791 (3)	175 (3)
C6—H6⋯O1^iii^	0.93	2.31	3.230 (3)	172
N2—H2*D*⋯O1^iii^	0.93 (5)	2.31 (5)	3.230 (3)	172 (4)
C5—H5⋯O4^iv^	0.93	2.38	3.282 (3)	165
O2—H2*B*⋯O4^iv^	0.73 (3)	2.02 (3)	2.739 (2)	172 (3)
O3—H3*A*⋯O1^v^	0.73 (3)	2.07 (3)	2.798 (3)	175 (3)

Symmetry codes: (iii) 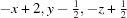; (iv) 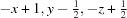; (v) 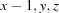.

**Table 3:** Experimental details

Crystal data	
Chemical formula	[Ni(C_6_H_6_N_2_O)_2_(H_2_O)_4_](C_4_H_2_O_4_)
*M*_r_	489.08
Crystal system, space group	Monoclinic, *P*2_1_/*c*
Temperature (K)	296
*a*, *b*, *c* (Å)	9.6140 (8), 9.9819 (9), 11.3874 (10)
β (°)	113.157 (7)
*V* (Å^3^)	1004.76 (16)
*Z*	2
Radiation type	Mo *K*α
μ (mm^−1^)	1.03
Crystal size (mm)	0.58 × 0.50 × 0.39

Data collection	
Diffractometer	STOE IPDS 2
Absorption correction	Integration (*X-RED32*; Stoe & Cie, 2002 ▸)
*T*_min_, *T*_max_	0.527, 0.593
No. of measured, independent and observed [*I* > 2σ(*I*)] reflections	5175, 2075, 1777
*R*_int_	0.045
(sin θ/λ)_max_ (Å^−1^)	0.628

Refinement	
*R*[*F* ^2^ > 2σ(*F* ^2^)], *wR*(*F* ^2^), *S*	0.041, 0.104, 1.05
No. of reflections	2075
No. of parameters	171
H-atom treatment	H atoms treated by a mixture of independent and constrained refinement
Δρ_max_, Δρ_min_ (e Å^−3^)	0.39, −0.83

Computer programs: *X-AREA* (Stoe & Cie, 2002 ▸), *X-RED* (Stoe & Cie, 2002 ▸), *SHELXL2017* (Sheldrick, 2015 ▸), *ORTEP-3 for Windows* (Farrugia, 2012 ▸), *WinGX* (Farrugia, 2012 ▸) and *PLATON* (Spek, 2009 ▸).

## supplementary crystallographic information

### Crystal data

**Table:**

[Ni(C_6_H_6_N_2_O)_2_(H_2_O)_4_](C_4_H_2_O_4_)	*F*(000) = 508
*M**_r_* = 489.08	*D*_x_ = 1.617 Mg m^−^^3^
Monoclinic, *P*2_1_/*c*	Mo *K*α radiation, λ = 0.71073 Å
*a* = 9.6140 (8) Å	Cell parameters from 8294 reflections
*b* = 9.9819 (9) Å	θ = 2.0–28.5°
*c* = 11.3874 (10) Å	µ = 1.03 mm^−^^1^
β = 113.157 (7)°	*T* = 296 K
*V* = 1004.76 (16) Å^3^	Prism, blue
*Z* = 2	0.58 × 0.50 × 0.39 mm

### Data collection

**Table:**

STOE IPDS 2 diffractometer	2075 independent reflections
Radiation source: sealed X-ray tube, 12 x 0.4 mm long-fine focus	1777 reflections with *I* > 2σ(*I*)
Detector resolution: 6.67 pixels mm^-1^	*R*_int_ = 0.045
rotation method scans	θ_max_ = 26.5°, θ_min_ = 2.8°
Absorption correction: integration (X-RED32; Stoe & Cie, 2002)	*h* = −12→10
*T*_min_ = 0.527, *T*_max_ = 0.593	*k* = −12→12
5175 measured reflections	*l* = −14→14

### Refinement

**Table:**

Refinement on *F*^2^	0 restraints
Least-squares matrix: full	Hydrogen site location: mixed
*R*[*F*^2^ > 2σ(*F*^2^)] = 0.041	H atoms treated by a mixture of independent and constrained refinement
*wR*(*F*^2^) = 0.104	*w* = 1/[σ^2^(*F*_o_^2^) + (0.073*P*)^2^ + 0.0362*P*] where *P* = (*F*_o_^2^ + 2*F*_c_^2^)/3
*S* = 1.05	(Δ/σ)_max_ < 0.001
2075 reflections	Δρ_max_ = 0.39 e Å^−^^3^
171 parameters	Δρ_min_ = −0.82 e Å^−^^3^

### Special details

**Table:**

Geometry. All esds (except the esd in the dihedral angle between two l.s. planes) are estimated using the full covariance matrix. The cell esds are taken into account individually in the estimation of esds in distances, angles and torsion angles; correlations between esds in cell parameters are only used when they are defined by crystal symmetry. An approximate (isotropic) treatment of cell esds is used for estimating esds involving l.s. planes.
Refinement. Refined as a 2-component inversion twin.

### Fractional atomic coordinates and isotropic or equivalent isotropic displacement parameters (Å^2^)

**Table:**

	*x*	*y*	*z*	*U*_iso_*/*U*_eq_	
Ni1	0.500000	0.500000	0.500000	0.02691 (15)	
O2	0.39639 (19)	0.36038 (17)	0.35626 (16)	0.0352 (3)	
O3	0.3976 (2)	0.65707 (16)	0.38085 (15)	0.0345 (3)	
O4	0.45031 (18)	0.63766 (14)	0.16741 (14)	0.0399 (4)	
O5	0.2761 (2)	0.47778 (18)	0.11437 (18)	0.0473 (4)	
O1	1.0888 (2)	0.70001 (16)	0.31656 (18)	0.0506 (4)	
N1	0.6822 (2)	0.52490 (16)	0.44312 (18)	0.0315 (4)	
C7	0.3828 (2)	0.5393 (2)	0.10101 (19)	0.0328 (4)	
C8	0.4341 (3)	0.4863 (2)	0.0015 (2)	0.0357 (5)	
N2	1.0342 (3)	0.5042 (3)	0.2095 (3)	0.0605 (7)	
C2	0.8968 (2)	0.5673 (2)	0.33931 (19)	0.0344 (4)	
C3	0.8503 (3)	0.6699 (2)	0.3963 (2)	0.0402 (5)	
H3	0.891089	0.755195	0.401464	0.048*	
C4	0.7431 (3)	0.6452 (2)	0.4456 (2)	0.0392 (5)	
H4	0.711583	0.715976	0.482316	0.047*	
C6	0.8353 (3)	0.4416 (2)	0.3374 (2)	0.0423 (5)	
H6	0.864249	0.369442	0.300562	0.051*	
C1	1.0136 (3)	0.5951 (2)	0.2856 (2)	0.0402 (5)	
C5	0.7303 (3)	0.4249 (2)	0.3910 (2)	0.0401 (5)	
H5	0.691168	0.339653	0.390746	0.048*	
H3B	0.402 (3)	0.655 (3)	0.312 (3)	0.048 (8)*	
H3A	0.318 (3)	0.670 (3)	0.369 (2)	0.038 (7)*	
H2B	0.434 (4)	0.297 (3)	0.354 (3)	0.051 (9)*	
H2A	0.364 (4)	0.389 (3)	0.287 (3)	0.056 (9)*	
H2C	1.030 (6)	0.554 (6)	0.139 (5)	0.133 (17)*	
H2D	0.989 (5)	0.420 (5)	0.199 (4)	0.104 (14)*	
H8	0.367 (3)	0.424 (3)	−0.058 (3)	0.053 (7)*	

### Atomic displacement parameters (Å^2^)

**Table:**

	*U*^11^	*U*^22^	*U*^33^	*U*^12^	*U*^13^	*U*^23^
Ni1	0.0289 (2)	0.0249 (2)	0.0340 (2)	0.00018 (13)	0.01994 (16)	−0.00068 (12)
O2	0.0414 (9)	0.0306 (8)	0.0386 (8)	0.0005 (7)	0.0210 (7)	−0.0031 (6)
O3	0.0366 (9)	0.0352 (8)	0.0389 (8)	0.0044 (7)	0.0226 (7)	0.0032 (6)
O4	0.0537 (10)	0.0327 (8)	0.0455 (8)	−0.0069 (7)	0.0328 (7)	−0.0049 (6)
O5	0.0479 (10)	0.0543 (10)	0.0529 (10)	−0.0138 (8)	0.0341 (8)	−0.0076 (7)
O1	0.0464 (10)	0.0421 (9)	0.0781 (12)	−0.0012 (7)	0.0405 (9)	0.0097 (8)
N1	0.0330 (9)	0.0291 (8)	0.0412 (9)	−0.0003 (7)	0.0241 (8)	0.0002 (7)
C7	0.0389 (11)	0.0291 (9)	0.0366 (9)	0.0038 (9)	0.0216 (9)	0.0031 (8)
C8	0.0425 (13)	0.0329 (11)	0.0393 (11)	−0.0024 (9)	0.0244 (10)	−0.0040 (8)
N2	0.0581 (16)	0.0683 (18)	0.0789 (18)	−0.0089 (12)	0.0526 (15)	−0.0146 (12)
C2	0.0274 (10)	0.0407 (12)	0.0397 (9)	0.0013 (9)	0.0182 (8)	0.0042 (9)
C3	0.0419 (12)	0.0314 (10)	0.0578 (12)	−0.0019 (9)	0.0309 (11)	0.0025 (9)
C4	0.0429 (12)	0.0305 (10)	0.0544 (12)	−0.0005 (9)	0.0302 (10)	−0.0024 (9)
C6	0.0422 (13)	0.0372 (12)	0.0592 (13)	−0.0031 (10)	0.0325 (11)	−0.0112 (10)
C1	0.0333 (11)	0.0469 (13)	0.0485 (11)	0.0040 (9)	0.0245 (10)	0.0086 (10)
C5	0.0424 (12)	0.0313 (11)	0.0584 (12)	−0.0042 (9)	0.0325 (11)	−0.0069 (9)

### Geometric parameters (Å, º)

**Table:**

Ni1—O3^i^	2.0536 (15)	C7—C8	1.499 (3)
Ni1—O3	2.0537 (16)	C8—C8^ii^	1.309 (5)
Ni1—O2	2.0812 (15)	C8—H8	0.95 (3)
Ni1—O2^i^	2.0812 (15)	N2—C1	1.322 (4)
Ni1—N1	2.1075 (18)	N2—H2C	0.93 (6)
Ni1—N1^i^	2.1075 (18)	N2—H2D	0.93 (5)
O2—H2B	0.73 (3)	C2—C3	1.378 (3)
O2—H2A	0.78 (3)	C2—C6	1.384 (3)
O3—H3B	0.80 (3)	C2—C1	1.501 (3)
O3—H3A	0.73 (3)	C3—C4	1.376 (3)
O4—C7	1.253 (3)	C3—H3	0.9300
O5—C7	1.255 (3)	C4—H4	0.9300
O1—C1	1.242 (3)	C6—C5	1.379 (3)
N1—C4	1.332 (3)	C6—H6	0.9300
N1—C5	1.334 (3)	C5—H5	0.9300
			
O3^i^—Ni1—O3	180.0	O5—C7—C8	116.5 (2)
O3^i^—Ni1—O2	88.00 (7)	C8^ii^—C8—C7	123.7 (3)
O3—Ni1—O2	92.00 (7)	C8^ii^—C8—H8	120.6 (17)
O3^i^—Ni1—O2^i^	92.00 (7)	C7—C8—H8	115.6 (16)
O3—Ni1—O2^i^	88.00 (7)	C1—N2—H2C	104 (4)
O2—Ni1—O2^i^	180.0	C1—N2—H2D	121 (3)
O3^i^—Ni1—N1	93.03 (7)	H2C—N2—H2D	121 (4)
O3—Ni1—N1	86.97 (7)	C3—C2—C6	117.72 (19)
O2—Ni1—N1	92.05 (6)	C3—C2—C1	119.23 (19)
O2^i^—Ni1—N1	87.95 (7)	C6—C2—C1	123.0 (2)
O3^i^—Ni1—N1^i^	86.97 (7)	C4—C3—C2	119.6 (2)
O3—Ni1—N1^i^	93.03 (7)	C4—C3—H3	120.2
O2—Ni1—N1^i^	87.95 (7)	C2—C3—H3	120.2
O2^i^—Ni1—N1^i^	92.05 (6)	N1—C4—C3	123.1 (2)
N1—Ni1—N1^i^	180.0	N1—C4—H4	118.4
Ni1—O2—H2B	121 (2)	C3—C4—H4	118.4
Ni1—O2—H2A	115 (2)	C5—C6—C2	119.0 (2)
H2B—O2—H2A	107 (3)	C5—C6—H6	120.5
Ni1—O3—H3B	116 (2)	C2—C6—H6	120.5
Ni1—O3—H3A	117 (2)	O1—C1—N2	122.9 (2)
H3B—O3—H3A	106 (3)	O1—C1—C2	119.0 (2)
C4—N1—C5	117.25 (18)	N2—C1—C2	118.0 (2)
C4—N1—Ni1	120.76 (14)	N1—C5—C6	123.3 (2)
C5—N1—Ni1	121.62 (14)	N1—C5—H5	118.4
O4—C7—O5	124.33 (18)	C6—C5—H5	118.4
O4—C7—C8	119.12 (18)		
			
O4—C7—C8—C8^ii^	17.1 (4)	C1—C2—C6—C5	178.9 (2)
O5—C7—C8—C8^ii^	−160.8 (3)	C3—C2—C1—O1	15.0 (3)
C6—C2—C3—C4	−1.7 (3)	C6—C2—C1—O1	−163.2 (2)
C1—C2—C3—C4	179.9 (2)	C3—C2—C1—N2	−166.6 (3)
C5—N1—C4—C3	0.6 (4)	C6—C2—C1—N2	15.1 (4)
Ni1—N1—C4—C3	−172.50 (19)	C4—N1—C5—C6	−1.9 (4)
C2—C3—C4—N1	1.2 (4)	Ni1—N1—C5—C6	171.21 (19)
C3—C2—C6—C5	0.6 (3)	C2—C6—C5—N1	1.3 (4)

Symmetry codes: (i) −*x*+1, −*y*+1, −*z*+1; (ii) −*x*+1, −*y*+1, −*z*.

### Hydrogen-bond geometry (Å, º)

**Table:**

*D*—H···*A*	*D*—H	H···*A*	*D*···*A*	*D*—H···*A*
O3—H3*B*···O4	0.80 (3)	1.89 (3)	2.678 (2)	169 (3)
O2—H2*A*···O5	0.78 (3)	2.01 (3)	2.791 (3)	175 (3)
C6—H6···O1^iii^	0.93	2.31	3.230 (3)	172
N2—H2*D*···O1^iii^	0.93 (5)	2.31 (5)	3.230 (3)	172 (4)
C5—H5···O4^iv^	0.93	2.38	3.282 (3)	165
O2—H2*B*···O4^iv^	0.73 (3)	2.02 (3)	2.739 (2)	172 (3)
O3—H3*A*···O1^v^	0.73 (3)	2.07 (3)	2.798 (3)	175 (3)

Symmetry codes: (iii) −*x*+2, *y*−1/2, −*z*+1/2; (iv) −*x*+1, *y*−1/2, −*z*+1/2; (v) *x*−1, *y*, *z*.
